# Positivity effect in source attributions of arousal-matched emotional and non-emotional words during item-based directed forgetting

**DOI:** 10.3389/fpsyg.2014.01334

**Published:** 2014-11-18

**Authors:** Sara N. Gallant, Lixia Yang

**Affiliations:** Department of Psychology, Ryerson UniversityToronto, ON, Canada

**Keywords:** item-based directed forgetting, source attributions, emotion, aging, positivity effect, memory

## Abstract

Consistent with their emphasis on emotional goals, older adults often exhibit a positivity bias in attention and memory relative to their young counterparts (i.e., a positivity effect). The current study sought to determine how this age-related positivity effect would impact intentional forgetting of emotional words, a process critical to efficient operation of memory. Using an item-based directed forgetting task, 36 young and 36 older adults studied a series of arousal-equivalent words that varied in valence (i.e., positive, negative, and neutral). Each word was followed by a cue to either remember or forget the word. A subsequent “tagging” recognition task required classification of items as to-be-remembered (TBR), to-be-forgotten (TBF), or new as a measure of directed forgetting and source attribution in participants' memory. Neither young nor older adults' intentional forgetting was affected by the valence of words. A goal-consistent valence effect did, however, emerge in older adults' source attribution performance. Specifically, older adults assigned more TBR-cues to positive words and more TBF-cues to negative words. Results are discussed in light of existing literature on emotion and directed forgetting as well as the socioemotional selectivity theory underlying the age-related positivity effect.

## Introduction

Research demonstrates a divergent trajectory for aging brains, characterized by declines in cognition (Salthouse, 2004) and preservation in emotional processing (Carstensen and Mikels, 2005). Although the ability to process emotional information appears to remain intact in late life, age differences often emerge with older relative to young adults, exhibiting a bias toward positive and/or away from negative information in both attention and memory (i.e., Reed and Carstensen, 2012). This phenomenon has been termed the *positivity effect* and is most often defined by three patterns of results: (1) a positivity bias in older but not young adults, (2) a negativity bias in young but not older adults, and (3) a co-occuring negativity bias in young adults and positivity bias in older adults (Langeslag and van Strien, 2009). According to the socioemotional selectivity theory (SST; Carstensen, 1995), older adults experience a shift in their time perspective as they approach the end of life. As a result, they exhibit an increased emphasis on emotional goals (e.g., emotion regulation, prioritization of meaningful relationships) than their young counterparts who may instead be more engaged with knowledge-related goals (e.g., obtaining a degree or promotion). It has therefore been proposed that older adults' age-related shift in emotional biases may be rooted in a motivation to satisfy emotional goals such as regulating emotion by seeking out positive and ignoring negative information (Reed and Carstensen, 2012). Prior findings support this theory by showing that older adults are selective in their information processing and invest more cognitive resources in the elaboration of positive stimuli, thus leading to better memory for positive relative to negative or neutral information (e.g., Charles et al., 2003). However, when those resources are depleted (e.g., by dividing attention), older adults' positivity bias often disappears (Mather and Knight, 2005; Reed et al., 2014), suggesting their bias may be rooted in effortful and resource demanding processes. Moreover, such age-related positivity effects in memory have shown to be specific to low-arousing emotional stimuli (Kensinger, 2008), which engage more controlled as opposed to automatic processes commonly involved in processing highly arousing stimuli (Kensinger and Corkin, 2004). Taken together, these findings demonstrate age-related positivity effects in attention and memory arising from an increased motivation to satisfy emotional goals. However, it is still unclear whether this positivity effect would impact instances where forgetting might be more favorable.

Although often perceived as maladaptive, forgetting is just as critical as our ability to remember. When intentional, forgetting benefits memory by deleting no-longer-needed material, thus releasing resources for the processing and manipulation of task-relevant information. In the laboratory, intentional forgetting is most often studied using the “directed forgetting” task in which participants are instructed to remember or forget certain types of information for a later memory test (Bjork, 1970; MacLeod, 1998). The two approaches to assess directed forgetting involve an item- and list-based task, which differ primarily in the timing and frequency of cue presentation (Basden and Basden, 1998). In the list-based version, a between-subjects design is typically employed in which two groups of participants study a list of stimuli. Following the list, each group receives a cue designating the list as either to-be-remembered (TBR) or to-be-forgotten (TBF). Both groups then study a second list, cued as TBR. In the item-based version, cues are presented following the presentation of each stimulus in a within-subjects manipulation. In both versions of the task, a subsequent memory test for all items is administered to evaluate the directed forgetting effect. In the list-based version, the effect is revealed when the group receiving the TBF cue remembers fewer items on the first list relative to the group receiving the TBR cue; in the item-based version, the effect is revealed when participants show greater memory for TBR relative to TBF items.

Studies assessing age differences in intentional forgetting have shown a significant directed forgetting effect in older adults, although the magnitude of their effect is generally smaller than young adults at least for neutral stimuli (Titz and Verhaeghen, 2010). So far, studies have only assessed the impact of emotion on directed forgetting in young adults, and so knowledge about age and emotion interactions in directed forgetting is missing. In this study, we focus on aging and emotion in the context of item-based directed forgetting, as emotional effects are more likely to be observed in intermixed lists of emotional and neutral stimuli than in conditions where stimuli of different emotional valence are presented in blocks (Dewhurst and Parry, 2000). In contrast to the block-based cue presentation in the list-based method, the item-based task facilitates use of intermixed lists as cues are delivered on an item-by-item basis, thus allowing for multiple condition comparisons within a large list of stimuli.

Studies of emotion in item-based directed forgetting of young adults, however, do not offer a clear-cut story. For example, prior investigations have found that when high-arousing negative images are compared against low-arousing neutral images in the item-based task, the directed forgetting effect is reduced or washed out for emotional but not neutral stimuli (e.g., Hauswald et al., 2010; Nowicka et al., 2011; Bailey and Chapman, 2012). Otani et al. (2011) also replicate this pattern of findings by showing reduced directed forgetting for high-arousing negative images relative to low-arousing neutral images, but coupled with significant directed forgetting of high-arousing positive images. Two recent investigations, however, diverge from this pattern of findings. On the one hand, Yang et al. (2012) showed comparable directed forgetting of arousal-matched negative and neutral images, suggesting that the arousal of image stimuli may be contributing to the previously observed disrupted directed forgetting of negative stimuli (e.g., Hauswald et al., 2010; Nowicka et al., 2011; Otani et al., 2011). On the other hand, Brandt et al. (2013) demonstrated comparable directed forgetting effects for high-arousing negative and low-arousing neutral *words*. This result suggests an important role of stimulus type in directed forgetting of emotional stimuli. These two recent findings (Yang et al., 2012; Brandt et al., 2013) also highlight another missing piece to the puzzle: does emotional valence, after controlling for arousal level, affect directed forgetting of emotional words? To fill this gap, the current study concerns item-based directed forgetting of emotional (positive and negative) and neutral words with equivalent levels of arousal. Together with earlier findings, the results will inform our understanding of the contributions of emotional valence (matched vs. mismatched at arousal level) and stimulus type (word vs. images) to item-based directed forgetting, while investigating the effects of age.

A secondary goal of the current study was to determine the effects of age and valence on source attributions and memory-binding efficiency (i.e., binding the cue and word at encoding) in the context of item-based directed forgetting. A recent investigation by Thompson et al. (2011) revealed the effectiveness of a “tagging” procedure in producing similar directed forgetting effects as a traditional old/new recognition tasks. In this procedure, a source (i.e., TBR, TBF, or new) is assigned to each word during the recognition test that follows encoding. This is different from an old/new recognition procedure in which TBR and TBF items are indistinguishably lumped into an “old” category. Thompson et al. (2011) posit that tagging items at recognition provides an added layer of information concerning the representation of TBR and TBF information in memory. For example, they provide evidence of a weaker memory trace for TBF items, as participants were more likely to confuse a new word as TBF as opposed to TBR during recognition, an effect that may be due to more effortful encoding devoted to TBR items during the study phase. In the context of item-based directed forgetting of emotional information, use of a tagging procedure such as this would provide information concerning emotional effects on memory representations of TBR and TBF items across young and older adults. For instance, perhaps due to their age-related bias toward positive information, older adults' memory traces for positive TBR and TBF items will be stronger relative to negative items? To our knowledge, only one other study has examined source attributions within the context of emotional item-based directed forgetting (Otani et al., 2011); however, source attributions were provided *following* the recall of all items, and thus may not accurately reflect the salience of memory trace for each item. In our study, we asked participants to tag items as TBR, TBF, or new on an item-by-item basis during recognition when the source of information is likely most accessible. As well, we extend the study of Otani et al.'s (2011) by examining age differences in source attribution of arousal-equivalent emotional and neutral words in item-based directed forgetting to better understand how age-related emotional biases might impact the memory representation of TBR and TBF items.

Thus, to address the above goals we asked young and older adults to complete an item-based directed forgetting task for arousal-equivalent positive, negative, and neutral words. This was followed by a tagging recognition procedure in which they were required to assign a TBR, TBF, or new tag to all previously studied words (i.e., TBR and TBF stimuli) and an equal amount of positive, negative, and neutral new words. Based on previous literature, we proposed several hypotheses. Concerning young and older adults' differential biases toward emotional information in attention and memory (Reed and Carstensen, 2012), it is reasonable to expect that emotion might differentially impact directed forgetting across the two age groups. For instance, directed by their emotional goals, older adults' tendency to process and remember positive information might make it particularly difficult to intentionally forget positive information relative to young adults, thus producing a positivity effect. It is also possible that young adults may show reduced directed forgetting for negative information relative to older adults (i.e., a negativity bias). Both of these results would support an age-associated positivity effect (Langeslag and van Strien, 2009), although the latter of the two seems less likely. Although a negativity bias has been observed with reduced directed forgetting of high-arousing negative relative to low-arousing neutral images (Otani et al., 2011), young adults have also demonstrated comparable item-based directed forgetting for high-arousing negative and low-arousing neutral words (Brandt et al., 2013). Thus, using arousal-matched emotional words, it is reasonable to hypothesize that young adults will show equivalent directed forgetting across valence categories as in Brandt et al. (2013). As such, a positivity effect rooted in older adults' positivity bias relative to young adults seems a more likely prediction. However, as the explicit directed forgetting instruction is cue-based and not valence-based, it might deplete the resources that older adults could otherwise use to engage in goal-consistent valence-based elaboration. Considering the absence of age-related positivity effects when goal-directed resources are divided (Mather and Knight, 2005; Reed et al., 2014), it is also possible that emotional valence might not produce an age-related positivity effect in directed forgetting at all.

Regarding general source attribution performance, an age-related decline was expected considering deficits in source memory with increasing age (McIntyre and Craik, 1987). However, since older adults have shown a memory advantage for emotional relative to non-emotional sources (e.g., May et al., 2005) and considering the incidental nature of the cue-word binding in the current task, valence might differentially affect older adults' source attributions relative to young adults. Specifically, guided by their goal-consistent intention to remember positive and forget negative information, older adults may be more likely than young adults to engage in goal-congruent processing to correctly or incorrectly attribute TBR cues to positive items and/or attribute TBF cues to negative items. Finally, consistent with Thompson et al. (2011), more TBF than TBR attributions to new words was predicted due to shallower encoding of TBF-cued stimuli during the directed forgetting task. This difference may diminish in older adults, as due to an inhibitory deficit in suppressing irrelevant information (Hasher and Zacks, 1988), they may be more likely than young adults to process the irrelevant TBF words, resulting in a stronger memory trace for TBF stimuli.

## Methods

### Participants

The final sample included 36 young (aged 18–28 years, 7 males) and 36 older adults (aged 65–85 years, 11 males). This sample size allowed for a high power (0.94) to detect the within-between interaction of even a small effect size (0.15) at α level of 0.05 (Faul et al., 2009). Young adults were recruited from the Introductory Psychology courses at Ryerson University. Older adults were recruited from the Ryerson Senior Participant Pool, which is largely made up of older adults who partake in the continuing education programs at Ryerson University. Young adults were compensated with a bonus course credit and older adults were paid $10 CAD for their participation.

We collected demographic information (e.g., age, sex, and education level) via a background questionnaire and administered socio-emotional and cognitive measures as a means to later control for potential confounds in the results (e.g., age differences in years of education). These measures included the Shipley Institute of Living vocabulary test (Shipley, 1946), Beck Anxiety Inventory (BAI; Beck et al., 1988), Center for Epidemiologic Studies Depression Scale (CES-D; Radloff, 1977), the Digit Symbol Substitution Test (DSST; Wechsler, 1981) to measure perceptual psychomotor speed and to complete during the delay between encoding and recognition, the Positive and Negative Affective Schedule (PANAS; Watson et al., 1988) to measure current mood, and the Short Blessed Test (SBT; Katzman et al., 1983) to test for potential cognitive impairment in the older sample. As typically found in cognitive aging literature (e.g., Charles et al., 2003; Yang and Ornstein, 2011; Truong and Yang, 2014), older adults were more educated, had higher vocabulary, slower psychomotor speed, higher positive and lower negative affect, and lower anxiety and depression scores (see Table 1). Age differences in these variables were later tested for their contribution to the main findings.

**Table 1:** **Characteristics of the final sample**.

	**Older (*n* = 36)**	**Younger (*n* = 36)**	
**Measure**	***M* (*SD*)**	***M* (*SD*)**	***p*-Value**
Age in years	71.53 (5.44)	20.22 (3.12)	0.000
Years of education	15.89 (2.11)	13.90 (2.79)	0.001
PANAS: positive affect	34.89 (8.43)	28.72 (7.84)	0.002
PANAS: negative affect	11.42 (3.53)	13.50 (4.88)	0.041
CES-D	7.42 (5.56)	16.61 (8.81)	0.000
BAI	3.25 (4.19)	13.08 (6.61)	0.000
Shipley vocabulary^a^	37.25 (1.71)	27.86 (3.56)	0.000
DSST^a^	58.81 (15.76)	86.00 (11.36)	0.000
SBT	0.53 (1.21)	–	–

a *score reflects number of correct solutions*.

Consistent with prior practices (e.g., Truong and Yang, 2014), we excluded and replaced participants based on criteria established a priori. As verbal English stimuli were used, participants were excluded if they learned English after age six or scored lower than 20 on the Shipley Institute of Living vocabulary test to ensure English language proficiency. To control for cognitive impairment, participants were excluded if they scored over the cut-off of six on the SBT. Based on findings that anxiety may elicit different emotional biases (Bradley et al., 1999; Dalgleish et al., 2003), participants were excluded if they scored over the cutoff of 26 on the BAI. We chose not to exclude participants based on depressive symptoms as CES-D scores have shown not to contribute to the age-related positivity effect, even when young adults score higher than older adults in depression on this measure (e.g., Charles et al., 2003). Thirteen young adults were excluded and replaced based on these criteria: three due to Shipley scores over 20, two for learning English after age six, and eight for scoring over 26 on the BAI suggesting severe anxiety symptoms. No participants were excluded based on SBT scores.

### Materials

The experiment was programmed with E-Prime 2.0 (Psychology Software Tools, Inc.). Stimuli were displayed in black lowercase Courier New size-18 font against a white background on a 17″ PC laptop with a viewing distance of approximately 60 cm.

#### Stimuli

A list of 120 positive, negative, and neutral words were selected from the Affective Norms for English Words (ANEW; Bradley and Lang, 1999) based on valence and arousal norms ranging from 1 (high negativity, low arousal) to 9 (high positivity, high arousal; see Table A1). Emotion conditions were significantly different on average valence (i.e., positive > neutral > negative), but matched on arousal, word length, and word frequency (see Table 2).

**Table 2:** **Characteristics of the selected word stimuli**.

	**Positive (*n =* 40)**		**Negative (*n =* 40)**		**Neutral (*n =* 40)**	
	***M* (*SD*)**	**Range**	***M* (*SD*)**	**Range**	***M* (*SD*)**	**Range**
Valence^a^	7.39 (0.40)	6.7–81	2.64 (0.61)	1.6–3.6	5.01 (0.55)	4.0–6.0
Arousal^b^	4.38 (0.63)	3.0–5.4	4.45 (0.51)	3.3–5.8	4.20 (0.40)	3.4–5.0
Length^c^	5.97 (0.26)	3.0–9.0	5.70 (0.25)	3.0–10.0	6.02 (0.26)	3.0–9.0
Frequency^d^	49.10 (47.02)	1.0–216.0	49.15 (64.80)	3.0–277.0	51.10 (69.30)	1.0–244.0

Ratings based on norms from the ANEW database (see Bradley and Lang, 1999). Valence categories:

a differed significantly on mean valence, ps < 0.001;

b matched on mean arousal, ps > 0.07;

c matched on mean word length, ps > 0.30; and

d *matched on mean word frequency, ps > 0.88*.

The list of 120 words was split into two sets of 60 with equal number of positive, negative, and neutral words. These sets were counterbalanced as “old” or “new” lists across participants during recognition. Each set was further evenly divided into two sub-sets of 30 words, each serving as to-be-remembered or to-be-forgotten stimuli, counterbalanced across participants. Each sub-set included 10 words from each valence. Lists were always matched on mean arousal, word length, and word frequency. Another 15 neutral words were selected, three used for practice and 12 as buffers.

### Procedure

All procedures were approved by the Ryerson University Research Ethics Board and conformed to regulatory standards in conducting psychological research. Participants were introduced to the experiment and informed consent was collected. They were asked to study a series of words and remember those followed by the cue “RRRR” and forget those followed by the cue “FFFF.” Participants were told that a memory test would follow, but were unaware that memory for TBF-cued words would be tested. As such, the cue-word binding process was incidental. After instructions, three practice trials were provided.

During encoding, participants completed 12 buffer trials (six at the beginning, six at the end) and 60 experimental trials (30 TBR, 30 TBF trials). Experimental trials were presented in a pseudo-randomized sequence, with no more than three of each valence or cue condition in a row. Each trial began with a fixation-cross at the screen's centre for 1000 ms, replaced by a word for 3000 ms. After the word, a blank screen appeared for 1500 ms as an inter-stimulus interval (ISI), followed by a memory cue (“RRRR” or “FFFF”) for 1000 ms, and another ISI for 500 ms. After encoding, participants completed the DSST for 2 min as a filler task, followed by a recognition test for 60 old and 60 new words. For each word, they were instructed to indicate as quickly and accurately as possible whether it was a TBR, TBF, or new word by pressing designated keys (“z,” “.,” or spacebar, labeled as “R,” “F,” and “NEW,” respectively). Trials were presented in a pseudo-randomized order, with no more than three words from each cue or valence condition occurring consecutively. Each trial consisted of a fixation-cross presented for 1000 ms at the center of the screen, and then replaced by a word that remained until a response was detected. Each response was followed by a 500 ms ISI.

At the end, participants completed the PANAS, Shipley, CES-D, BAI, SBT (older adults only), and a background questionnaire. Finally, they were debriefed and compensated.

## Results

All statistical analyses were conducted using IBM SPSS Statistics 19.0, with α levels set at 0.05, unless specified otherwise. Greenhouse-Geisser corrections were used where necessary.

### Recognition performance

**Figure 1:**
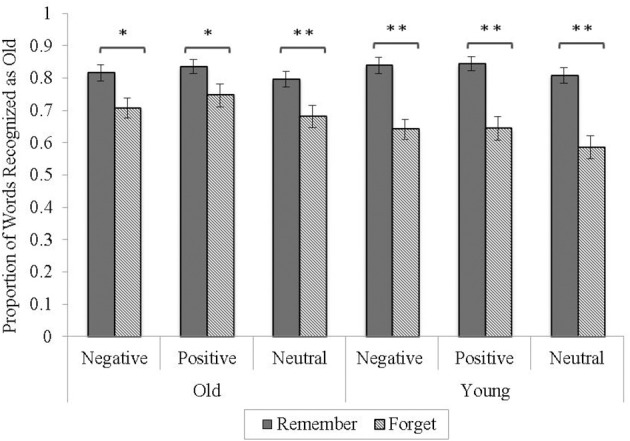
**Proportion of items recognized as “old” as a function of age, valence, and word type**. Error bars represent standard errors of the means. ^*^p < 0.01, ^**^p < 0.001.

Following prior procedures (Thompson et al., 2011), all “TBR” and “TBF” responses to each word type (TBR, TBF, and new) were combined to provide an overall item-based index of “old” responses (e.g., old responses to TBR-cued items = TBR responses to TBR-cued items + TBF responses to TBR-cued items). This provided an overall hit rate to each of the TBR- and TBF-cued items and a false alarm rate to new items. Hit rates were then analyzed in a 2 (age: young, old) × 3 (valence: negative, positive, neutral) × 2 (word type: TBR, TBF) mixed-model analysis of variance (ANOVA) with age as the only between-subjects variable (see Figure 1).

The analysis revealed a significant directed forgetting effect (i.e., main effect of word type), *F*_(1, 70)_ = 75.39, *p* < 0.001, η^2^_*p*_ = 0.52, with greater recognition of TBR-cued (*M* = 0.82, *SD* = 0.11) than TBF-cued items (*M* = 0.67, *SD* = 0.18), *t*_(71)_ = 8.28, *p* < 0.001. An effect of valence also emerged, *F*_(2, 140)_ = 5.70, *p* < 0.01, η^2^_*p*_ = 0.10. Follow-up analysis showed a higher recognition of positive (*M* = 0.77, *SD* = 0.15) than neutral items (*M* = 0.71, *SD* = 0.15), *t*_(71)_ = 3.40, *p* < 0.01; and a marginally higher recognition of negative (*M* = 0.75, *SD* = 0.13) than neutral items, *t*_(71)_ = 1.95, *p* = 0.06. However, positive and negative items did not differ in recognition, *p* > 0.20.

The age by word type interaction was also significant, *F*_(1, 70)_ = 8.10, *p* < 0.01, η^2^_*p*_ = 0.10. Follow-up *t*-tests showed that young adults had reduced recognition of TBF-cued items (*M* = 0.62, *SD* = 0.19) relative to older adults (*M* = 0.71, *SD* = 0.15) *t*_(70)_ = 2.17, *p* = 0.05, but no age difference between recognition TBR-cued items was observed (old, *M* = 0.82, *SD* = 0.10; young, *M* = 0.83, *SD* = 0.11), *t*_(70)_ = −0.55, *p* > 0.60. Taken together, this interaction suggests young adults had greater forgetting of TBF items and/or older adults had reduced capacity to inhibit processing of TBF items (i.e., reduced directed forgetting in older adults). All other main effects and interactions were non-significant, *p*s > 0.30.

False alarm rates were also submitted to a 2 (age) × 3 (valence) mixed-model ANOVA. The results revealed only a significant valence effect, *F* = 19.17, *p* < 0.001, η *_p_*^2^ = 0.22. Follow-up tests showed a higher false alarm rate for positive (*M* = 0.34, *SD* = 0.22) than negative words (*M* = 0.30, *SD* = 0.20), *t*_(71)_ = −2.75, *p* < 0.01; and both positive and negative showed a higher false alarm rate relative to neutral words (*M* = 0.25, *SD* = 0.21), *t*s > 3.60, *p*s < 0.01. All other main effects and interactions were non-significant, *p*s > 0.70.

### Source attribution performance

**Figure 2:**
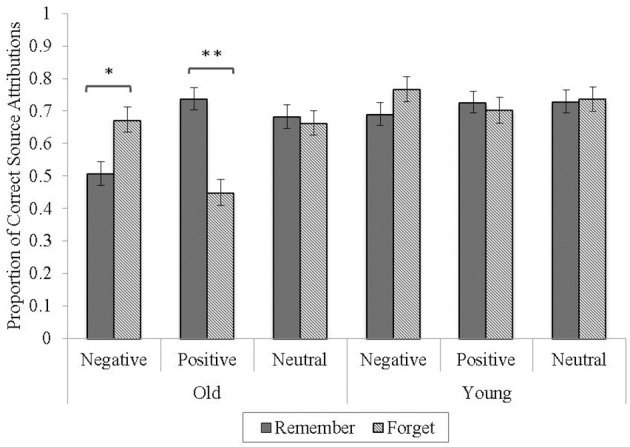
**Proportion of correct source attributions as a function of age, valence, and word type**. Error bars represent standard errors of the means. ^*^p < 0.05, ^**^p < 0.01.

Following previous practice in Thompson et al. (2011), correct source attribution performance was indexed as the proportion of old words attributed to a correct source out of the total number of old items correctly recognized as “old” for each valence. For example, the source attribution of the TBR-cued items was calculated as the “TBR” responses to TBR-cued items / (“TBR” responses to TBR-cued items + “TBF” responses to TBR-cued items). The source attribution data were then submitted to a 2 (age) × 2 (word type) × 3 (valence) mixed-model ANOVA (see Figure 2).

The analysis yielded a main effect of age, *F*_(1, 70)_ = 23.24, *p* < 0.001, η *_p_*^2^ = 0.25, with better source attribution performance in young (*M* = 0.73, *SD* = 0.11) than older adults (*M* = 0.62, *SD* = 0.08). Importantly, this 73% accuracy rate in young adults is consistent with that of Thompson et al.'s (2011) finding of 75% accuracy. All other main effects were non-significant (*p*s > 0.07).

The valence by word type interaction was significant, *F*_(2, 140)_ = 16.00, *p* < 0.001, η *_p_*^2^ = 0.19, and qualified by a three-way age by valence by word type interaction, *F*_(2, 140)_ = 6.69, *p* < 0.01, η *_p_*^2^ = 0.09. Follow-up ANOVAs revealed a significant valence by word type interaction for older, *F*_(1.64,57.28)_ = 16.25, *p* < 0.001, η *_p_*^2^ = 0.32, but not young adults, *p* > 0.20 (see Figure 2). Interestingly, older adults had reduced source accuracy for TBF-cued (*M* = 0.45, *SD* = 0.27) relative to TBR-cued positive items (*M* = 0.74, *SD* = 0.22), *t*_(35)_ = 4.10, *p* < 0.001, an effect that was reversed for negative words, with lower accuracy for TBR-cued (*M* = 0.51, *SD* = 0.23) than TBF-cued items (*M* = 0.67, *SD* = 0.24), *t*_(35)_ = −2.57, *p* < 0.05 in older adults. However, their accuracy did not differ between TBR-cued (*M* = 0.68, *SD* = 0.21) and TBF-cued neutral items (*M* = 0.66, *SD* = 0.23), *p* > 0.70. Valence did not impact young adults' source attributions.

Following from the results above, we sought evidence for whether older adults' source attribution performance was due to a response bias at recognition or differential encoding-based processes. Using the two-high threshold model (Snodgrass and Corwin, 1988), we computed response bias scores for each valence and cue combination using the calculation: *B*_r_ = False alarms / [1 − (Hits − False alarms)]. Scores equal to 0.5 indicate a neutral bias, above 0.5 indicate a conservative bias, and below 0.5, a liberal bias. Older adults' scores were all less than 0.5 (*M* = 0.18), indicating an overall liberal response bias. These scores were submitted to a 2 (word type) × 3 (valence) ANOVA. A valence by word type interaction, *F*_(2, 70)_ = 15.11, *p* < 0.001, η *_p_*^2^ = 0.30, suggested that the response bias to each word type varied by valence category. Specifically, for the TBR-cued items, older adults showed less liberal biases for negative (*M* = 0.40, *SD* = 0.60) than positive (*M* = −0.17, *SD* = 0.55), *t*_(35)_ = 4.258, *p* < 0.001, or neutral items (*M* = −0.10, *SD* = 0.46), *t*_(35)_ = 3.80, *p* < 0.01. In contrast, for the TBF-cued items, they showed a less liberal bias for positive (*M* = 0.47, *SD* = 61) than negative (*M* = −0.01, *SD* = 0.50), *t*_(35)_ = −3.91, *p* ≤ 0.001, or neutral items (*M* = 0.01, *SD* = 0.43), *t*_(35)_ = 3.47, *p* < 0.01. Together, these findings seem to correspond to older adults' source attribution performance with reduced source accuracy for positive TBF- and negative TBR-cued items and suggest their performance may have been partially due to response bias during recognition.

Next, following Thompson et al. (2011), source misattributions to new items were calculated as TBR or TBF attributions to new items out of all new items misrecognized as “old” (e.g., for TBR misattributions to new items: “TBR” responses to new items / [“TBR” responses to new items + “TBF” responses to new items]). The data of four young and four older participants who recognized all new items correctly as new were excluded from this analysis (to ensure the performance of these participants did not influence any other results, we re-ran the main recognition and correct source attribution analysis while excluding their data; no significant changes were observed to any of the main findings). Overall, new items were assigned more TBF-tags (*M* = 0.70, *SD* = 22) than TBR-tags (*M* = 0.30, *SD* = 0.22), *t*_(63)_ = −7.44, *p* < 0.001.

**Figure 3:**
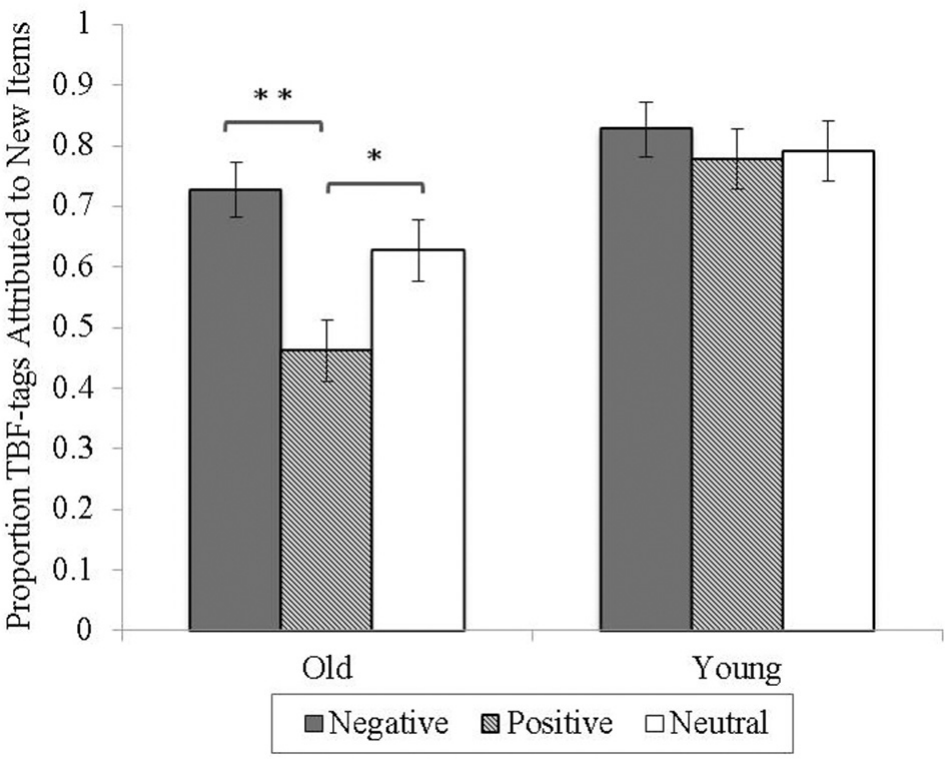
**Proportion of TBF-tags assigned to new items recognized as “old” as a function of age and valence**. Error bars represent standard errors of the means. p^*^ < 0.01, ^**^p < 0.001.

To determine age and valence effects on misattributions to new items, a 2 (age) × 3 (valence) ANOVA was conducted on the proportion of new items assigned a TBF-tag. TBR-tags to new items were excluded, as they are simply the inverse of TBF-tags to new items. The analysis revealed a main effect of age, *F*_(1, 62)_ = 15.61, *p* < 0.001, η *_p_*^2^ = 0.20, with young adults (*M* = 0.80, *SD* = 0.19) assigning more TBF-tags to new items than older adults (*M* = 0.61, *SD* = 0.20). There was also a main effect of valence, *F*_(2, 124)_ = 7.28, *p* < 0.01, η *_p_*^2^ = 0.11, qualified by an age by valence interaction, *F*_(2, 124)_ = 3.63, *p* < 0.05, η *_p_*^2^ = 0.06. Here, older adults assigned fewer TBF-tags to positive than negative, *t*_(31)_ = 3.88, *p* < 0.001, or neutral new items, *t*_(31)_ = −3.17, *p* < 0.01. There was no difference between TBF-tag assignment to negative and neutral new items. Young adults' assignment of TBF-tags to new items was unaffected by valence, *p*s > 0.34 (see Figure 3).

### Potential confounding variables

As several age differences were observed in the demographic, socio-emotional, and cognitive measures collected (see Table 1), it was important to determine if any of these group differences could account for our main findings. As such, correlations were run between each of the measures showing significant age differences and the dependent variables in the recognition, correct source attribution, and misattribution analyses across age groups. No significant relationships were observed between any of the collected measures and the dependent variables.

## Discussion

This study sought to investigate the effects of age and valence on intentional forgetting and source attributions using an item-based directed forgetting task and tagging recognition procedure (Thompson et al., 2011). Results indicate that both age groups can intentionally forget words from each valence condition when matched on arousal. However, the ability to assign TBR or TBF sources to words during recognition appears to be affected by valence and this effect varies across age groups, with emotional valence affecting older but not young adults. Results thus extend existing literature on age differences in attention and memory for emotional information (e.g., Reed and Carstensen, 2012) to source attributions in the context of item-based directed forgetting.

### Directed forgetting

Findings from the recognition task replicated the directed forgetting effect with better recognition of TBR than TBF items (MacLeod, 1998). This directed forgetting effect was reduced in older adults, driven by young adults' greater success in implementing a cue to forget. This effect implies that older adults are not as effective at suppressing the processing of TBF stimuli relative to young adults, which is consistent with prior research on age differences in item-based directed forgetting. For example, in a meta-analysis by Titz and Verhaeghen (2010), older adults generally showed an item-based directed forgetting effect, although it was reliably smaller than that of young adults. This pattern of findings also supports inhibitory deficit hypotheses of aging suggesting reduced capacity to suppress irrelevant information in late life (Hasher and Zacks, 1988).

Despite overall greater recognition of emotional relative to neutral words, neither older nor young adults' directed forgetting was affected by valence. We predicted a positivity effect here defined by a disruption to directed forgetting of positive information in older relative to young adults (Langeslag and van Strien, 2009); however, this hypothesis was not fulfilled. The absence of a positivity effect seemingly goes against the SST, which posits an age-related emphasis on emotional goals (Carstensen, 1995) shown to affect cognition (Reed and Carstensen, 2012). Following this line of reasoning, one might expect older adults to experience difficulty following a forget instruction for positive words, but this was not the case in our investigation. The findings are, however, consistent with observations of null positivity effects in contexts that are goal-irrelevant and involve resource-consuming task instructions that direct attention away from valence-based processing of items (e.g., Mather and Knight, 2005; Reed et al., 2014). For example, in a recent large-scale meta-analysis, Reed et al. (2014) found that the positivity effect was absent when constraints (e.g., divided attention) were placed on information processing, reducing the available resources for goal-consistent processing. Thus, when older adults' chronically activated emotional goals are overridden by the demands of a task, the goal to prioritize positive information is no longer fulfilled. Applying this to the present experiment, the directed forgetting task instructions to commit or forget items in memory for a later test, may have interfered with older adults' emotional goals, thus resulting in absence of an age by valence interaction in directed forgetting.

In addition, young adults did not show an emotional effect. This result is also at odds with several investigations reporting disruption in directed forgetting where high-arousing emotional images are compared against a low-arousing neutral baseline (Hauswald et al., 2010; Nowicka et al., 2011; Otani et al., 2011). It is consistent, however, with the findings of Yang et al. (2012) who show equivalent forgetting of arousal-matched negative and neutral pictures and those of Brandt et al. (2013) who report equivalent forgetting of high-arousing negative and low-arousing neutral words. Our results also add a novel finding to the literature by suggesting that emotional valence of word stimuli, when arousal-matched, appears not to disrupt directed forgetting. Taken together, it appears that arousal may mediate the valence effect on item-based directed forgetting for images, but not for words, in young adults. That is, in young adults, disrupted directed forgetting has been observed for emotional images that are more arousing than their neutral baseline, but significant directed forgetting when emotional and neutral images are matched on arousal. In comparison, directed forgetting of words may not be affected by emotional valence, regardless of arousal. Inability to forget high-arousing emotional images (e.g., of a tragic car accident or of a gun pointing at the viewer) may represent an adaptive quality in humans. Emotional arousal often signifies to an individual that a specific event or stimulus may become relevant for survival (Hamann, 2001). As such, following an instruction to purge such information from memory may not be adaptive to an individual, thus resulting in dissociation in directed forgetting of high-arousing emotional pictures and words. We acknowledge, however, that this dissociation is very speculative and warrants further research.

### Source attributions

Replicating Thompson et al.'s (2011) 75% source attribution accuracy with young adults, we found a comparable 73% accuracy rate in our young participants, promoting the utility of this procedure in future investigations. Consistent with expectations and previous findings (McIntyre and Craik, 1987), source attribution accuracy was reduced in older adults (62%). Furthermore, source attributions of older but not young adults were affected by valence. Relative to their young counterparts, older participants showed differentially better source attributions for positive TBR- than positive TBF-cued words and for negative TBF- than negative TBR-cued words. This result suggests that older adults' memory trace may have been stronger for positive TBR-cued and negative TBF-cued items. This is consistent with the SST as cues to remember positive and forget negative words are consistent with their emotional goals, whereas cues to forget positive and remember negative words may have conflicted with their emotional goals. This, in turn, reduces their ability to bind a TBF-cue to positive words and a TBR-cue to negative words.

That valence effects were observed in older adults' source attributions and not their item recognition may again be attributable to context-dependent positivity effects (see Reed et al., 2014). The act of binding the cue and word together during encoding was incidental, meaning that participants were not explicitly instructed to do so. In contrast, the directed forgetting instruction was explicit and intentional. As a result, older adults' emotional goals may have been more influential to their performance in the less resource-disruptive incidental binding of the cue and word during encoding. Consequently, their source attribution performance was reduced for goal-incongruent TBF-cued positive and TBR-cued negative items. However, the finding of a less liberal bias for each of the TBF-cued positive and TBR-cued negative conditions also suggests that older adults' performance may have been partially due to a bias that made them less likely to attribute each of these stimuli to their correct sources. That is, these higher bias scores relative to the other cue-valence combinations suggest that older adults were less willing to guess TBF positive and TBR negative items as belonging to their correct respective sources (Snodgrass and Corwin, 1988). As such, it is clear that older adults' emotional goals may have biased their responses, making them less likely to attribute a “forget” cue to their preferred positive information and conversely a “remember” cue to their less-preferred negative information. Further research is warranted to clarify whether these effects are due to enhanced binding of words and goal-congruent cues during encoding or strictly to a response bias during recognition.

Finally, consistent with predictions, a greater proportion of TBF- than TBR-tags were assigned to new words. This effect was most likely due to weaker memory traces for TBF-cued items after engaging in directed forgetting that caused participants to confuse new items with stimuli cued as TBF. As hypothesized, older adults' assignment of TBF-tags to new words was further reduced relative to young adults, presumably due to young adults' greater ability to suppress processing of TBF-cued stimuli. Moreover, an interaction between age and valence was observed, with older adults showing reduced attribution of TBF-tags to positive relative to negative or neutral new words. This finding implies that older adults may have a preference to say they remember a positive word, even in instances where they are unsure (i.e., when attributing sources to new information).

### Limitations

It should be noted that in our sample, young adults' average CES-D scores were above the cut-off of 16, suggesting sub-threshold depressive symptoms. Despite research showing depression could impact emotional directed forgetting (Power et al., 2000), no correlations were found between CES-D scores and directed forgetting across valence conditions in young adults. Therefore, it is unlikely that CES-D scores influenced our findings. The second limitation of the current study is that it offers little information about the exact mechanism of the source attribution performance. As mentioned previously, it is unclear whether the valence effects observed in older adults' source attribution were due to encoding or retrieval based processes. Further research using neuroimaging or neurophysiological techniques may help to further pinpoint when and how valence exerts its effect on source attributions in older adults in the context of directed forgetting.

## Conclusions

Methodologically, these results support the utility of Thompson et al.'s (2011) tagging procedure and extend its use to an older adult population. This procedure is advantageous as it provides additional information concerning the memory representation of TBF and TBR items during recognition. For example, our results indicate that participants were more likely to guess a new word as TBF rather than TBR, consistent with the assumption that directed forgetting induces shallower encoding and a consequently weaker memory trace for TBF-cued items. As a result, participants are more likely to confuse a TBF item with a new word. These cue-related differences would not be captured by the traditional old/new recognition paradigm.

From a theoretical standpoint, the findings suggest that while older adults' memory and attention is affected by the valence of stimuli (e.g., Charles et al., 2003), their intentional forgetting appears not to vary across emotional valence of arousal-matched words. Nevertheless, their overall directed forgetting is reduced relative to young adults. These findings have significant implications, considering the importance of intentional forgetting for efficient memory processing (MacLeod, 1998). Moreover, results shed light on our understanding of the differential roles of arousal in directed forgetting depending on the type of emotional stimuli utilized (i.e., words or images). Finally, older adults were differentially better at attributing sources to goal-consistent information: positive TBR-cued or negative TBF-cued stimuli. These findings add to the literature by suggesting that older adults may have an advantageous memory trace for sources of information that are tailored to their goals (i.e., to remember positive and forget negative).

## Author contributions

Both authors contributed to the design of the experiment. Sara N. Gallant led data acquisition, data analysis, and drafted the manuscript. Lixia Yang edited and critiqued the draft. Both authors approved the final version of the manuscript.

### Conflict of interest statement

The authors declare that the research was conducted in the absence of any commercial or financial relationships that could be construed as a potential conflict of interest.

## Appendices

**Table A1:** **Stimuli selected from ANEW (Bradley and Lang, 1999) database**.

**Negative**	**Positive**	**Neutral**
Alone	Angel	Alley
Blind	Bath	Aloof
Blister	Beauty	Appliance
Cemetery	Bed	Black
Coward	Bird	Blase
Cut	Bless	Board
Dead	Breeze	Cannon
Death	Brother	Coarse
Discomfort	Cake	Contents
Dummy	Carefree	Context
Failure	Comfort	Corner
Fat	Dream	Corridor
Fault	Elegant	Custom
Feeble	Gentle	Dark
Fever	Grateful	Errand
Gloom	Heal	Excuse
Grief	Hug	Gender
Handicap	Leisurely	Habit
Ignorance	Luxury	Haphazard
Illness	Melody	Knot
Immature	Music	Lump
Impair	Nature	Material
Inferior	Ocean	Medicine
Lost	Pillow	Muddy
Messy	Politeness	Obey
Moody	Protected	Odd
Neglect	Rainbow	Passage
Obesity	Respectful	Patient
Poverty	Reward	Privacy
Rat	Safe	Quart
Sad	Satisfied	Rock
Scar	Secure	Sheltered
Sick	Sky	Shy
Slum	Snuggle	Spray
Stink	Soft	Stagnant
Stupid	Sunset	Stiff
Trash	Twilight	Stomach
Unhappy	Useful	Tower
Useless	Warmth	Trumpet
Waste	Wise	Writer
